# Inhibition of alpha7 nicotinic receptors in the ventral hippocampus selectively attenuates reinstatement of morphine‐conditioned place preference and associated changes in AMPA receptor binding

**DOI:** 10.1111/adb.12624

**Published:** 2018-04-17

**Authors:** Victoria L. Wright, Polymnia Georgiou, Alexis Bailey, David J. Heal, Christopher P. Bailey, Susan Wonnacott

**Affiliations:** ^1^ Department of Biology & Biochemistry University of Bath UK; ^2^ Department of Pharmacy & Pharmacology University of Bath UK; ^3^ Faculty of Health and Medical Sciences University of Surrey UK; ^4^ Department of Psychiatry University of Maryland School of Medicine Baltimore MD USA; ^5^ Institute of Medical and Biomedical Education St George's University of London UK; ^6^ RenaSci UK

**Keywords:** [^3^H]‐AMPA, [^3^H]‐MK801, autoradiography, intracerebral drug delivery, methyllycaconitine

## Abstract

Recurrent relapse is a major problem in treating opiate addiction. Pavlovian conditioning plays a role in recurrent relapse whereby exposure to cues learned during drug intake can precipitate relapse to drug taking. α7 nicotinic acetylcholine receptors (nAChRs) have been implicated in attentional aspects of cognition and mechanisms of learning and memory. In this study we have investigated the role of α7 nAChRs in morphine‐conditioned place preference (morphine‐CPP). CPP provides a model of associative learning that is pertinent to associative aspects of drug dependence. The α7 nAChR antagonist methyllycaconitine (MLA; 4 mg/kg s.c.) had no effect on the acquisition, maintenance, reconsolidation or extinction of morphine‐CPP but selectively attenuated morphine‐primed reinstatement of CPP, in both mice and rats. Reinstatement of morphine‐CPP in mice was accompanied by a selective increase in [^3^H]‐AMPA binding (but not in [^3^H]‐MK801 binding) in the ventral hippocampus that was prevented by prior treatment with MLA. Administration of MLA (6.7 μg) directly into the ventral hippocampus of rats prior to a systemic priming dose of morphine abolished reinstatement of morphine‐CPP, whereas MLA delivered into the dorsal hippocampus or prefrontal cortex was without effect. These results suggest that α7 nAChRs in the ventral hippocampus play a specific role in the retrieval of associative drug memories following a period of extinction, making them potential targets for the prevention of relapse.

## Introduction

Drug addiction is a chronic relapsing brain disorder (Leshner 1997; Koob & Volkow 2010), with relapse posing the greatest obstacle to overcoming addiction. For example, almost 3 million people in the United States are estimated to be addicted to prescription opiates or heroin (National Survey on Drug Use and Health, 2013; Lyapustina & Alexander 2015). Despite the availability of substitution products such as methadone, relapse to heroin use is prevalent. Relapse may be triggered by a variety of stimuli including drug‐associated cues, exposure to sub‐threshold (priming) doses of drug or stress (Shalev, Grimm, & Shaham 2002). Understanding the brain mechanisms underlying the different stages of drug addiction may identify novel targets for intervention.

The cholinergic system is intimately engaged with reward circuitry in the brain. Nicotine, acting through nicotinic acetylcholine receptors (nAChRs) within these circuits, sustains tobacco addiction (Wonnacott, Sidhpura, & Balfour 2005; Brunzell, Stafford, & Dixon 2015), which commonly co‐exists with the use of other addictive substances. Indeed, tobacco smoking has been regarded as a gateway to illicit drug use (Jessup 1996; Kandel & Kandel 2015). Physiologically, nAChRs mediate the actions of endogenous acetylcholine, which is implicated in attention (notably to cues that prompt goal‐directed behaviours), motivation and learning and memory, important facets of addiction (Luchicchi *et al*. 2014; Sarter *et al*. 2014). Thus, nAChRs may contribute to general processes underlying drug dependence and nAChRs have been proposed as novel targets for treating addiction (Rahman, Engleman, & Bell 2015).

The α7 nAChR, which is highly expressed in the hippocampus and cortex (Yakel 2014), has gained prominence for its association with attentional aspects of cognition and mechanisms of learning and memory (Levin, Hall, & Rezvani 2015). In the present experiments, we sought to investigate the contribution of α7 nAChRs to opiate reward, using the conditioned place preference (CPP) model of motivational learning, with morphine as the unconditional stimulus. This model is well established (Bardo & Bevins 2000; Tzschentke 2007; Aguilar, Rodríguez‐Arias, & Miñarro 2009) and allows acquisition, maintenance, extinction and reinstatement of drug‐seeking behaviour to be interrogated. A previous report (Feng *et al*. 2011) has implicated α7 nAChRs in morphine‐primed reinstatement of CPP in mice, but other components of morphine‐seeking behaviour were not examined. Therefore, we have used the selective antagonist methyllycaconitine (MLA), delivered systemically and intracerebrally, to block α7 nAChRs at different stages of morphine‐CPP. Changes in glutamate receptor density were monitored in parallel, using quantitative autoradiography, in order to shed light into the possible mechanisms underlining these effects. This study has revealed a selective action of α7 nAChR antagonism in the ventral hippocampus that inhibits both morphine‐primed CPP and associated increases in AMPA receptor binding in this brain region.

## Materials and Methods

### Animals

Male C57BL/6 mice (6–7 weeks of age at the start of experiments; Charles River, Kent, UK) and Wistar rats (400 g, University of Bath breeding colony) were housed in groups of four, except following surgery when rats were singly housed, in a controlled environment [12:12 hour light–dark cycle (lights on 07:00); constant temperature (24 ± 2°C) and humidity (55 ± 5%)]. Food and water were available *ad libitum*. Animals were handled daily for 1 week prior to the start of experiments. All experiments were approved by the UK Home Office and performed in accordance with UK Animals (Scientific Procedures) Act of 1986 and conform to the Animals in Research: Reporting In Vivo Experiments (ARRIVE) guidelines (Kilkenny *et al*. 2010).

### Drugs

Morphine hydrochloride was purchased from MacFarlan Smith, Edinburgh, UK; MLA was purchased from Tocris Cookson, Avonmouth, UK. For peripheral administration, drugs were dissolved in sterile saline and injected in a volume of 10 ml/kg. Control animals received vehicle injections (sodium chloride 0.9% *w*/*v*, Hameln Pharmaceuticals, Gloucester, UK). For intracerebral injections, MLA (2.8 mg/ml) was dissolved in saline. [^3^H]‐(+)‐MK801 (22.5 Ci/mmol) and [^3^H]‐AMPA (58.1 Ci/mmol) were from Perkin Elmer Life Sciences, USA. CNQX, (+)‐MK801 and all other reagents were from Sigma, Poole, Dorset, UK.

### Conditioned place preference procedure

Conditioned place preference (CPP) was carried out essentially as previously described (Ribeiro Do Couto *et al*. 2003; Cordery *et al*. 2014). Mouse CPP was in two‐compartment shuttle boxes (Ugo Basile, Gemonio, Italy). Compartments were 15 cm square; one compartment consisted of grey walls and a metal floor with circular holes, the other had striped walls and square holes. Rat CPP was in three‐compartment shuttle boxes (Tracksys, Nottingham, UK). Compartments were 40 cm square, linked via a 10‐cm square central area; one compartment consisted of horizontal black and white stripes and a metal floor with circular holes, the other had vertical black and white stripes and a metal floor with square holes.

Experiments were performed between 08:00 and 17:00 under dim white light (light intensity approximately 15 lux). During all preference test sessions (typically 15 minutes), the animal had free access to both compartments; the time each animal spent in each compartment and their locomotor activity (distance travelled) was recorded using EthoVision XT version 8.0 (Tracksys) tracking software.

#### Acquisition of morphine‐conditioned place preference

**Figure 1:**
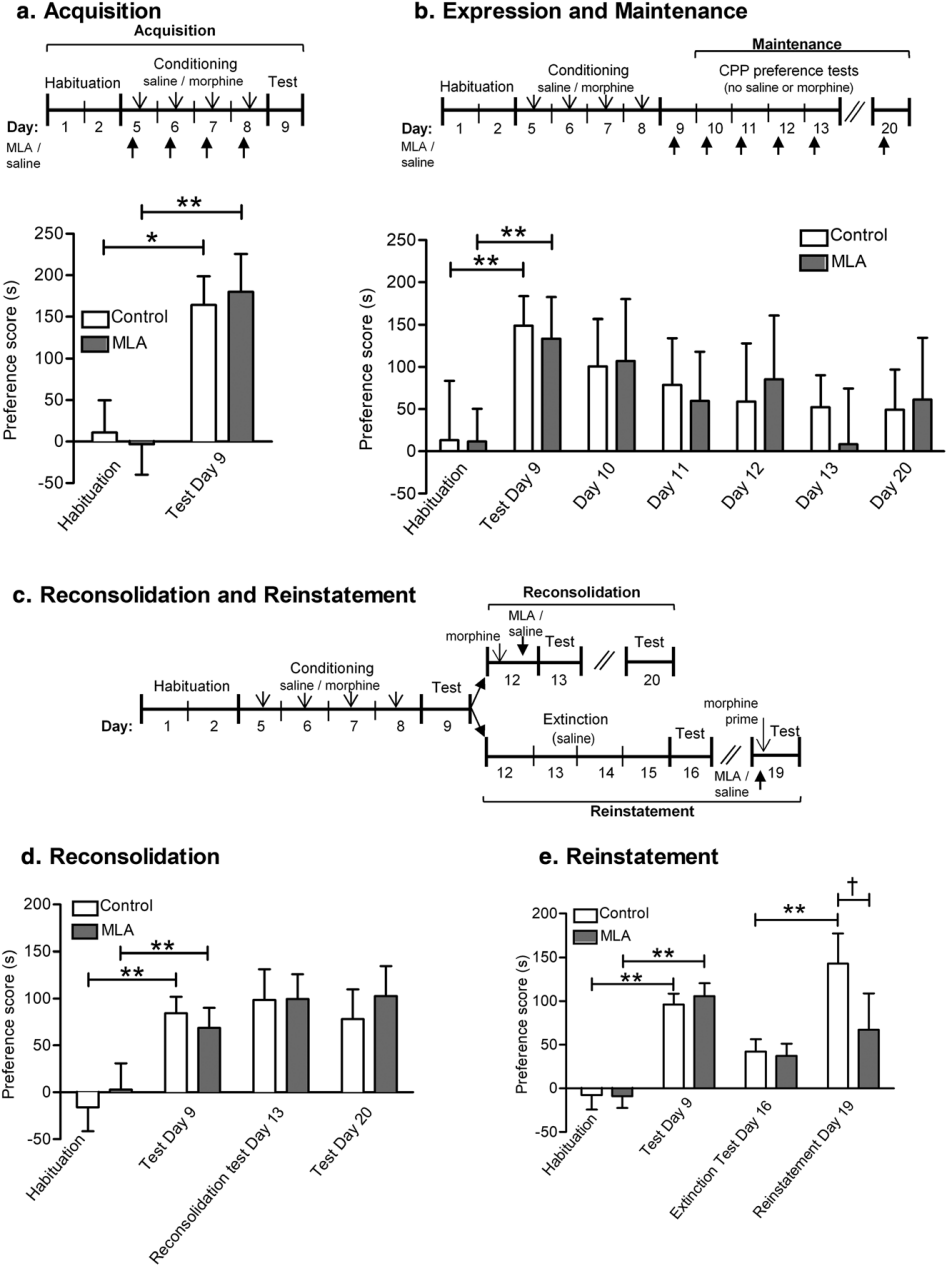
Effect of MLA on stages of morphine‐CPP in C57BL/6 mice. (a) Acquisition. Mice were tested for any innate preference for a particular chamber (Habituation) and then pseudo‐randomly allocated to two groups with comparable mean preference scores. The MLA group received MLA (4 mg/kg) 20 minutes prior to a conditioning dose of morphine (10 mg/kg) or saline, paired with alternative compartments, on 4 consecutive days. The control group received saline instead of MLA. A preference test was conducted on the next day, giving the animals free access to both compartments of the CPP apparatus for 15 minutes. Preference scores indicate the time spent in the morphine‐paired compartment in seconds minus 450 (half of the total time). Data are expressed as the mean ± S.E.M. Both groups showed significant acquisition of morphine‐CPP (*saline: *P* < 0.05, **MLA: *P* < 0.005, *n* = 16 per group) indicating that MLA treatment had no effect on the acquisition of morphine‐CPP. (b) Maintenance. Mice were allocated to two groups and both groups were treated identically, to acquire morphine‐CPP as in (a). They were tested for expression of morphine‐CPP on 5 consecutive days and 1 week later. One group received MLA (4 mg/kg) 20 minutes prior to each preference test, the control group received saline instead of MLA. Both groups acquired morphine‐CPP (**P < 0.005, *n* = 12 per group). MLA treatment had no effect on the expression of morphine‐CPP during the maintenance phase. (c) Schematic for reconsolidation and reinstatement experiments. (d) Reconsolidation. Two groups of mice were treated identically, to acquire morphine‐CPP as in (a). Three days later mice received a further dose of morphine (10 mg/kg) in the drug‐paired compartment. Immediately afterwards, one group received MLA (4 mg/kg) while the control group received saline. The mice underwent a preference test the next day and one week later. Both groups acquired morphine‐CPP (**P < 0.005, *n* = 12 per group). Their preference for the drug‐paired compartment was consolidated by morphine and unaffected by MLA. E. Reinstatement. Two groups of mice were treated identically, to acquire morphine‐CPP as in (a), followed by 4 days of extinction, (saline injections only, paired with alternative compartments) on separate days. Both groups acquired morphine‐CPP (**P < 0.005, *n* = 20 per group) and this was attenuated following extinction training. On the following day, mice received a priming dose of morphine (5 mg/kg) prior to an extended preference test (30 minutes); one group received MLA (4 mg/kg) 20 minutes before morphine; the control group received saline instead of MLA. Only the control group of mice showed significant reinstatement of morphine‐CPP in contrast to MLA‐treated mice (saline: **P < 0.005). The time spent in the morphine‐paired compartment was significantly different between MLA and saline treatments (^†^ *P* < 0.005). MLA significantly inhibited reinstatement of morphine‐CPP

Following drug‐free habituation trials (1 × 15‐minute session/day for 2 days, with free access to both compartments) to reveal any innate preference for either of the compartments, animals were pseudorandomly assigned to treatment groups that were organized so that mean baseline preferences for a particular compartment were close to zero (Fig. 1a). Morphine conditioning consisted of 1 × 40‐minute trial/day for 4 days, starting 2 days after the habituation test. Each animal was confined to one compartment following injection of morphine (mice: 10 mg/kg i.p.; rats 5 mg/kg s.c.) or saline. The drug/saline pairing and compartment were reversed on consecutive days, so that each animal received two morphine injections and two saline injections. A counterbalanced design was employed such that within a treatment group, half the animals were drug‐paired with one compartment type and the other half were drug‐paired with the other compartment type; the order of morphine or saline presentation was also counterbalanced. A drug‐free post‐conditioning preference test was carried out 24 hour after the last drug treatment. Animals were placed in the CPP box with free access to both compartments for 15 minutes and time spent in each compartment was recorded, to determine their preferences. Saline‐only controls were included in a preliminary experiment to validate the established protocol (Fig. S1). Thereafter, all subjects received morphine in the counterbalanced design described, to test the effects of α7 nAChR antagonism.

To examine the effect of systemic MLA on morphine‐CPP acquisition, animals received MLA (4 mg/kg s.c.) or saline 20 minutes before each conditioning dose of morphine.

#### Maintenance of morphine‐conditioned place preference

To assess whether MLA affected the maintenance of morphine‐CPP, animals were subjected to morphine conditioning as described previously (Fig. 1b). Following the post‐conditioning preference test to establish that they had acquired morphine‐CPP, they were subjected to additional preference tests over the following 4 days and 1 week later. MLA (4 mg/kg) or saline was given 20 minutes before each test.

#### Reconsolidation of morphine‐conditioned place preference

Animals acquired morphine‐CPP as described earlier (Fig. 1d). Three days after the post‐conditioning preference test, all animals received morphine and were immediately confined to their drug‐paired side of the CPP box for 40 minutes (reconsolidation step). MLA (4 mg/kg) or saline was administered at the end of this session, prior to return to the home cage. A preference test was carried out 1 day and 7 days later.

#### Extinction and reinstatement of morphine‐conditioned place preference

Animals acquired morphine‐CPP, as described previously, followed by extinction training (Figs 1e, 4 & 5). Animals received saline injections only, paired with alternate compartments of the CPP box, over 4 consecutive days. On the following day, the animals were subjected to another preference test. Only those that met criterion (less than 70% of time spent in the previously drug‐paired compartment) were considered to show extinction and proceeded to the reinstatement phase. Fewer than 5% of subjects were excluded.

Three days later, for the reinstatement of morphine‐CPP, animals received a priming dose of morphine (mice: 5 mg/kg i.p.; rats 2.5 mg/kg s.c.) prior to being given an extended preference test (30 minutes, based on a preliminary experiment), with free access to both compartments of the CPP box; the time spent in each compartment was monitored over two 15 minutes bins. Preliminary data showed development of reinstatement over the 30‐minute test session (as in Mueller, Perdikaris, & Stewart 2002), therefore the second 15 minutes bin was taken for reinstatement values. MLA (4 mg/kg s.c.) or saline was administered 20 minutes before the priming dose of morphine. In mice, saline‐only controls were included in a preliminary experiment to validate the established protocol (Fig. S1). Reinstatement produced more variable data sets, requiring a larger sample size for statistical power.

### Intracerebral cannula implantation and drug delivery

Bilateral cannulae were implanted into the medial PFC (mPFC), ventral or dorsal hippocampus of 52 rats following acquisition and extinction of morphine‐CPP, but prior to the reinstatement test. Rats were anaesthetized with isoflurane (induction 4%, maintenance, 2–3%, Baxter, UK), placed into a stereotaxic frame and guide cannulae were implanted into the mPFC (coordinates relative to bregma: anterior–posterior +3.2, medial–lateral ±0.75, dorsal–ventral −2.8), the dorsal hippocampus (anterior–posterior −3.2, medial–lateral ±2.5, dorsal–ventral −1.8) or ventral hippocampus (anterior–posterior −5.3, medial–lateral ±5.2, dorsal–ventral −4.5). Dummy cannulae were inserted and secured with a dust cap to prevent post‐surgical infection. The rats were rehydrated with 0.9% saline solution (10 ml/kg s.c.) and were given an antibiotic (0.2 ml clamoxyl LA, 150 mg/ml s.c.) and an analgesic (Caprieve, 5 mg/kg s.c). Animals were allowed to recover for 7 days.

Infusions were performed in conscious, gently restrained animals 15 minutes before the reinstatement trial. MLA or saline (2.4 μl /hemisphere) was delivered over 4 minutes (0.6 μl/min) via a 33 gauge infusion cannula, using an infusion pump (Harvard apparatus). The cannulae were left in place for a further 4 minutes. Infusions were conducted in pairs of animals.

To verify cannula placement, at the end of the experiment, rats were killed by rising CO_2_ asphyxiation, and 0.5‐ml brilliant blue dye was infused via each cannula, using the infusion pump. Brains were frozen in isopentane and stored at −80°C. Coronal sections were made using a cryostat at −21°C. Sections containing the dye marks were compared with a brain atlas (Paxinos & Watson 2007; Fig. S2). Three animals were culled due to adverse reaction to surgery; no animals were excluded following verification of cannula placement.

### Quantitative autoradiography

Twenty‐four mice underwent morphine‐CPP acquisition followed by extinction training (Fig. 1c) and were then randomly assigned to one of the four groups:

- Saline pretreatment/saline reinstatement (6)
- MLA pretreatment/saline reinstatement (6)
- Saline pretreatment/morphine‐primed reinstatement (6)
- MLA pretreatment/morphine‐primed reinstatement (6)

Animals were killed by cervical dislocation immediately after the reinstatement test, brains were frozen in isopentane and stored at −80°C. Adjacent 20 μm coronal sections were collected containing the prefrontal cortex (bregma +1.94 mm), striatum (bregma +1.42 mm), dorsal hippocampus (bregma −1.22 mm) and the mid‐brain/ventral hippocampus (bregma −3.08 mm; Paxinos & Franklin 2012). Consecutive sections were freeze‐thaw mounted onto separate gelatin‐subbed glass slides for total and non‐specific labelling and processed for autoradiography as described previously (Kitchen *et al*. 1997).

#### N‐methyl‐d‐aspartate receptor quantitative autoradiography

Slides were preincubated (20 minutes, 20°C) in 50 mM Tris‐HCl, pH 7.4, containing 50‐μM glutamate, 50‐μM glycine and 50‐μM spermidine. Subsequently, slides were incubated (1 hour, 4°C) in the same buffer containing 70 nM [^3^H]‐(+)‐MK801 to determine total binding. Non‐specific binding was determined in the presence of 1 mM (+)‐MK801 (Reynolds 2001). Slides were rinsed twice for 30 seconds in ice‐cold 50 mM Tris‐HCl, pH 7.4, followed by distilled water and rapidly dried with a stream of cool air and placed in a sealed container with anhydrous calcium sulphate for 1 week.

#### AMPA receptor quantitative autoradiography

AMPA receptors were labeled with [^3^H]‐AMPA as previously described (Duncan *et al*. 2002). Slides were pre‐incubated (20 minutes, 20°C) in 50 mM Tris‐HCl, pH 7.4, containing 50 mM sodium thiocyanate. Total binding was determined by incubating sections (45 minutes, 20°C) in the same buffer containing 10 nM [^3^H]‐AMPA. Non‐specific binding was determined in adjacent sections incubated in the additional presence of 0.1 mM CNQX. Slides were rinsed three times for 20 seconds in ice‐cold 50 mM Tris‐HCl, pH 7.4, followed by distilled water and rapidly dried with a stream of cool air and stored with anhydrous calcium sulphate as for [^3^H]‐(+)‐MK801 binding.

Slides were apposed to photographic film (Kodak Biomax MR‐1, Sigma‐Aldrich, UK) along with autoradiographic [^3^H] microscale standards (GE Healthcare, Amersham, U.K.) for 3 weeks ([^3^H]‐AMPA) or 4 weeks ([^3^H]‐(+)‐MK801). Sections for all treatment groups were processed in parallel and apposed to the same film at the same time; slides were developed and analysed in parallel (Kitchen *et al*.^1997^); structures were identified by reference to the mouse brain atlas of Franklin & Paxinos (2001) and analysed using an image analyser (MCID; Image Research, Linton, UK). Specific binding was determined by subtracting non‐specific binding from total binding: for [^3^H]‐AMPA labelling, non‐specific binding was homogeneous; therefore, a representative non‐specific binding area was subtracted from all total values; for [^3^H]‐(+)‐MK801 labelling, non‐specific binding was taken from the corresponding area for each brain region analysed.

### Data analysis

All data are presented as mean ± standard error of the mean (S.E.M). CPP data are presented as Preference Scores for the time (seconds) spent in morphine‐paired compartment; these are calculated as time spent in morphine‐paired compartment−450 (half the maximum time, in seconds). All behavioural analyses were performed using *in vivo stat* with a one‐way ANOVA with repeated measures, with *post hoc* analysis with Benjamini–Hochberg test for multiple comparisons when significant interaction effects were observed (*P* < 0.05). For analysis of the effects of intracerebral MLA, a one‐way ANOVA was performed. For analysis of autoradiographic binding, a two‐way ANOVA with factors ‘treatment’ (saline versus MLA) and ‘behaviour’ (saline‐primed versus morphine‐primed reinstatement) was performed for each region analysed.

## Results

### Effect of the α7 nicotinic acetylcholine receptor antagonist methyllycaconitine on morphine‐conditioned place preference in mice

Mice readily acquired morphine‐CPP and displayed a significant preference for the compartment associated with the morphine dose (10 mg/kg i.p.) after 4 days of conditioning (Fig. S1). This could be extinguished by pairing saline treatments with both compartments. Morphine‐CPP could be reinstated by a priming dose of morphine (5 mg/kg i.p.) in mice that had acquired morphine‐CPP prior to extinction (Fig. S1). The conditioning and priming doses of morphine were based on previously published studies in mice (Tzschentke 2007; Feng *et al*. 2011) and pilot studies to optimize priming dose.

The involvement of α7 nAChRs in morphine‐CPP was examined by systemic administration of MLA (4 mg/kg s.c.). This dose of MLA was based on literature evidence for its selectivity and efficacy in mice (Feng *et al*. 2011). MLA itself did not induce any CPP or aversion (MLA + saline: habituation 3.7 ± 28.6 seconds, post conditioning 33.1 ± 40.7 seconds versus saline + saline: habituation 18.3 ± 52.9 seconds, post conditioning 16.2 ± 41.0 seconds, no effect of treatment *P* = 0.75 or test *P* = 0.45, *n* = 7/treatment group; data not shown).

MLA was administered at different stages of morphine‐CPP (either prior to morphine administration, prior to testing or immediately after a morphine‐conditioned reconsolidation trial; Fig. 1a–c, respectively). MLA given prior to morphine or saline before each conditioning trial did not affect acquisition of morphine‐CPP (Fig. 1a). There was no significant effect of treatment (*F*(_1,22_) = 0.15, *P* = 0.699) but a significant effect of test (*F*(_1,30_) = 24.09, *P* = <0.001). *Post hoc* pairwise comparisons revealed significant morphine‐CPP in saline + morphine group (preference score during habituation: 10.8 ± 39.0 seconds; post‐conditioning: 164.1 ± 34.5 seconds, *n* = 16, *P* = 0.012), and MLA + morphine group (preference score during habituation: −3.2 ± 36.7 seconds; post‐conditioning: 179.8 ± 45.8 seconds, *n* = 16, *P* = 0.003). There was no significant difference between the MLA pretreated and the saline pretreated groups (*n* = 16, *P* = 0.69).

To assess if α7 nAChRs have any effect on the maintenance and expression of morphine‐CPP, mice underwent morphine conditioning followed by a post‐conditioning preference test and were again tested for their preference scores on the following 4 days and 1 week later. MLA was given 20 minutes before each preference test (Fig. 1b). CPP was significantly learned in both treatment groups: there was a significant effect of test (*F*(_6,78_) = 4.37, *P* = 0.001, *n* = 12/treatment) but not of treatment (*F*(_1,13_) = 0.78, *P =* 0.394). *Post hoc* pairwise comparisons indicated significant expression of morphine‐CPP on preference test day 1 (saline: *P* = 0.004, MLA: *P* = 0.024, *n* = 12/treatment). Preference scores declined only slowly over time with repeated testing; importantly, there was no significant difference at any time‐point between saline and MLA‐treated animals, showing that MLA did not affect the expression and maintenance of morphine‐CPP.

To determine if α7 nAChRs play any role in the reconsolidation of morphine‐CPP, MLA was given immediately after a reconsolidation trial (in which morphine, 10 mg/kg, was paired with the previously drug‐paired compartment; Fig. 1c). Mice were tested for their compartment preference 24 hours and 7 days later (Fig. 1d). There was no significant effect of MLA treatment (*F*(_1,17_) = 0, *P =* 0.961) but a significant effect of test (*F*(_2,44_) = 14.25, *P =* <0.001, *n* = 12/treatment group). *Post hoc* analysis revealed no significant difference between treatment groups after 24 hours or 1 week later indicating that MLA had no effect on reconsolidation of morphine‐induced CPP.

Next, MLA was tested on reinstatement of morphine‐CPP: mice acquired morphine‐CPP which was then extinguished by repeated saline administration in association with both compartments of the CPP apparatus; MLA or saline was given prior to a priming dose of morphine (5 mg/kg) to reinstate morphine‐CPP (Fig. 1c). Immediately, after receiving the priming dose, mice were tested in the CPP apparatus for any compartment preference. The priming dose of morphine produced a robust reinstatement of morphine‐CPP whereas prior treatment with MLA significantly attenuated reinstatement (Fig. 1e). There was no significance in the effect of treatment (*F*(_1,52_) = 1.15, *P =* 0.288) but a significant effect of test (*F*(_3,156_) = 13.48, *P =* <0.001, *n* = 20/treatment group). *Post hoc* analysis for multiple comparisons revealed that only animals that received saline prior to the priming dose of morphine showed significant reinstatement (saline preference score at extinction: 42.2 ± 14.0 seconds versus 143.1 ± 33.2 seconds at reinstatement; *P* = 0.003; MLA preference score at extinction: 37.3 ± 13.8 seconds versus 67.3 ± 41.7 seconds at reinstatement, *P* = 0.135, *n* = 20/treatment group). The time spent in the drug paired side after morphine‐primed reinstatement was significantly different between the two treatments (*P* = 0.0016).

### Glutamate receptor binding: effect of reinstatement of morphine‐conditioned place preference and methyllycaconitine pretreatment

**Figure 2:**
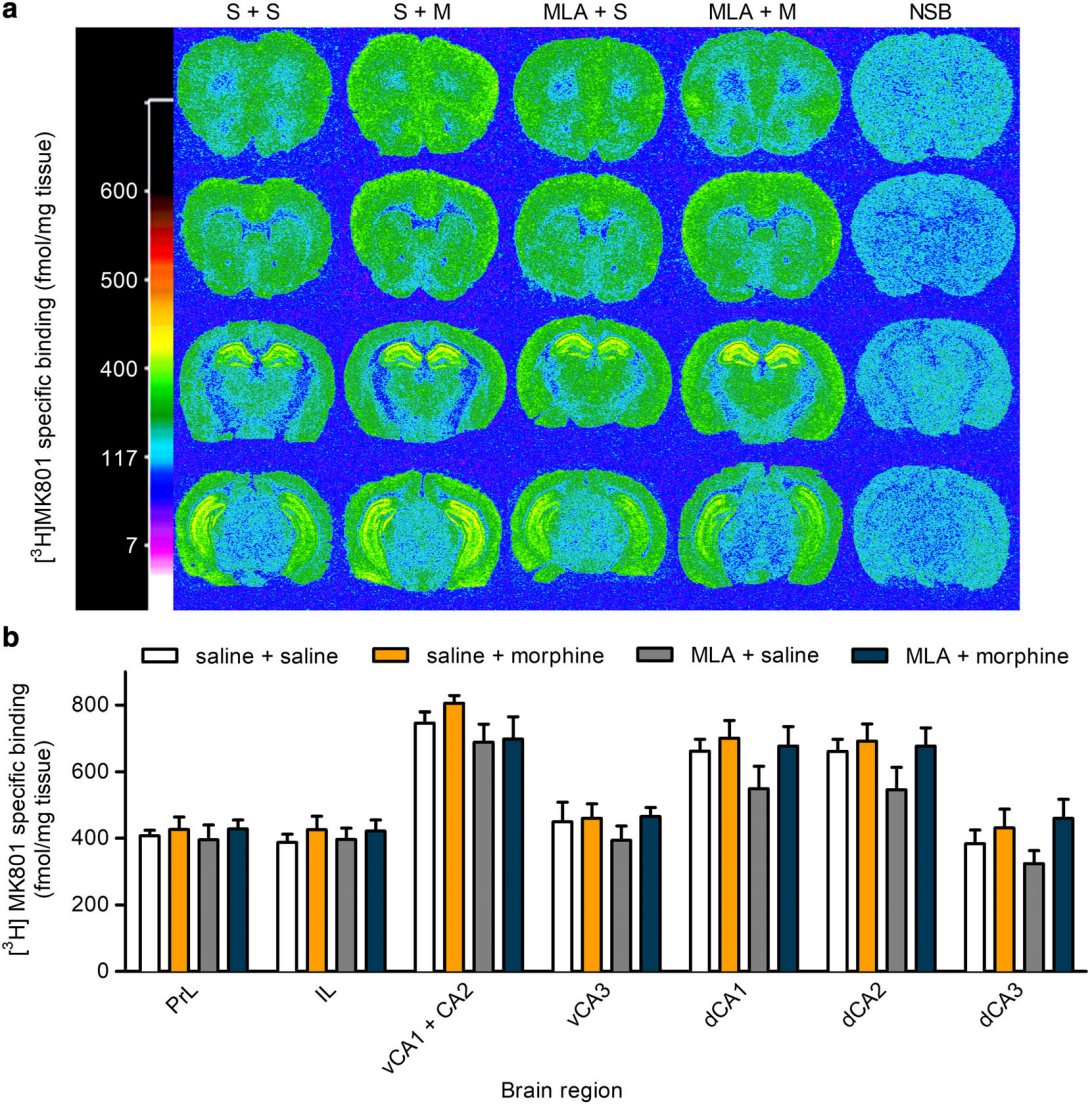
Effect of morphine‐CPP reinstatement, in the presence and absence of MLA, on NMDA receptor binding in mouse brain. Animals underwent morphine‐CPP acquisition and extinction (Fig. 1c). A parallel set of animals followed the same procedure but received saline instead of morphine. Each group was then randomly divided into two sets that received either saline or MLA (4 mg/kg) 20 minutes before saline or a priming dose of morphine (5 mg/kg) and were then tested for reinstatement of morphine‐CPP. Immediately afterwards, mice were killed and brains were prepared for autoradiography as described in the Methods. Sections were labelled with 70 nM [^3^H]‐(+)‐MK801, in the absence and presence of 1 μM (+)‐MK801 to determine non‐specific binding. (a) Representative computer‐enhanced autoradiograms of [^3^H]‐(+)‐MK801 binding in coronal brain sections of mice taken at four levels corresponding to prefrontal cortex (bregma 1.94 mm), striatum (bregma 1.42 mm), dorsal hippocampus (bregma −1.22 mm) and the midbrain/ventral hippocampus (bregma −3.08 mm; Paxinos & Frankilin 2012). Binding levels are represented using a pseudocolour interpretation of black and white film images in fmol/mg tissue equivalent. Representative autoradiograms for non‐specific binding (NSB) are shown (far right column). (b) Quantitative NMDA receptor binding. Levels of [^3^H]‐(+)‐MK801 binding (fmol/mg tissue equivalent) are shown for prelimbic cortex (PrL), infralimbic cortex (IL), CA1 and CA2 regions of the ventral hippocampus combined (vCA1 + CA2), ventral hippocampus CA3 (vCA3), dorsal hippocampus CA1, CA2 and CA3 separately (dCA1, dCA2 and dCA3), for each of the treatment groups (saline + saline control, saline + morphine‐primed reinstatement, MLA + saline control, MLA + morphine‐primed reinstatement). Data are presented as mean ± S.E.M. (*n* = 4–7). There were no statistically significant differences between any treatment groups in any of the brain regions quantified

Quantitative autoradiography was used to determine the levels of [^3^H]‐(+)‐MK801 and [^3^H]‐AMPA binding in brain sections prepared from the brains of mice that had undergone reinstatement of morphine‐CPP, or saline‐primed controls, with or without MLA pretreatment. Sections were taken at four levels from Bregma. Representative autoradiographs of [^3^H]‐(+)‐MK‐801 binding are shown in Figure 2a. There was differential labelling of brain regions, with highest levels in the hippocampus, followed by cortical areas, in agreement with previous reports (Bowery, Wong, & Hudson 1988). There was no difference in specific [^3^H]‐(+)‐MK‐801 binding between treatment groups, in any of the brain regions analysed (Fig. 2b; Table S1).

**Figure 3:**
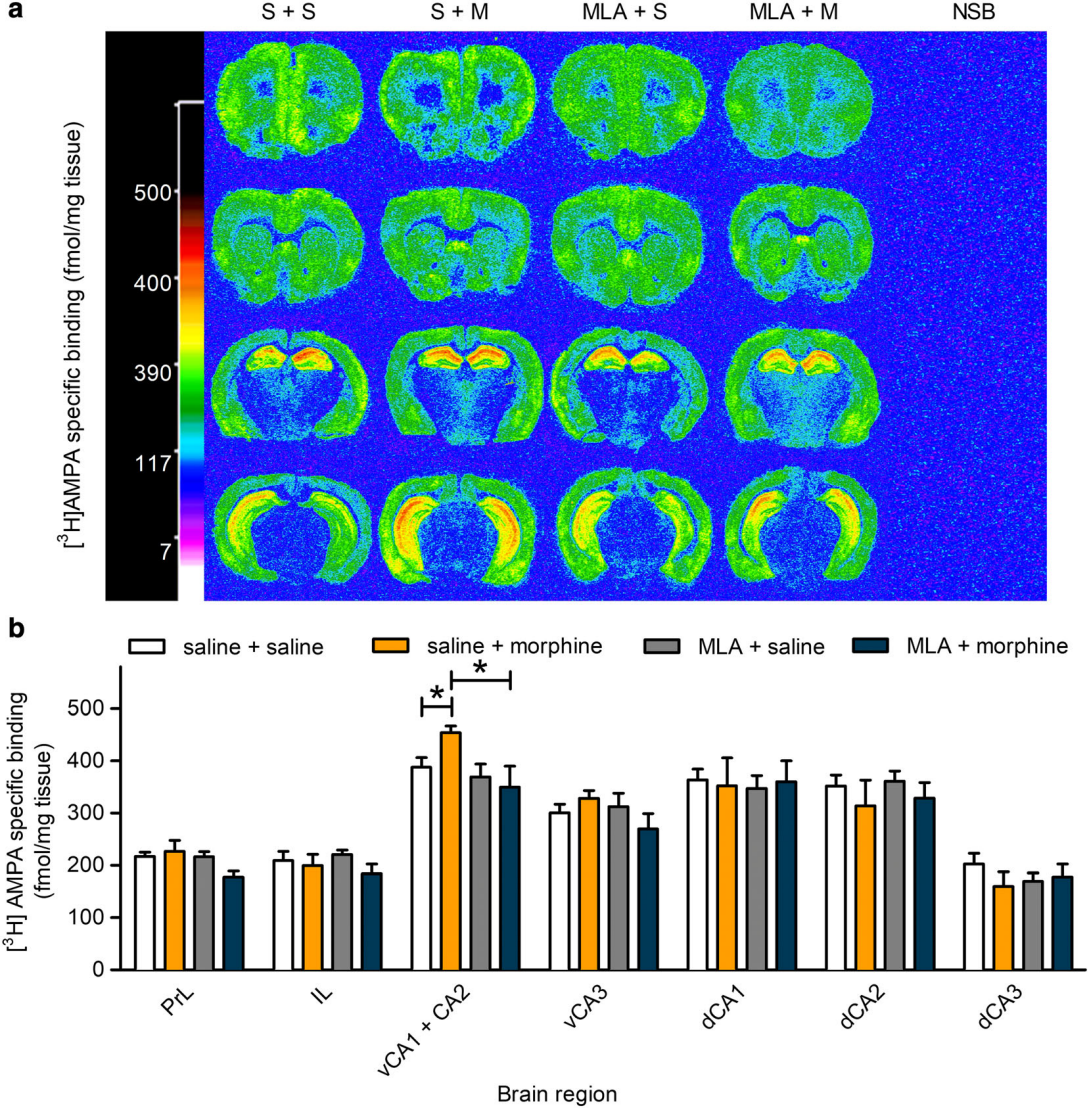
Effect of morphine‐CPP reinstatement, in the presence and absence of MLA, on AMPA receptor binding in mouse brain. Animals underwent morphine‐CPP acquisition and reinstatement, and their brains were prepared for autoradiography as described in the legend to Figure 2. Sections were labelled with 10 nM [^3^H]‐AMPA, in the absence and presence of 0.1 μM CNQX to determine non‐specific binding. (a) Representative computer‐enhanced autoradiograms of [^3^H]‐AMPA binding in coronal brain sections of mice taken at four levels corresponding to prefrontal cortex (bregma 1.94 mm), striatum (bregma 1.42 mm), dorsal hippocampus (bregma −1.22 mm) and the midbrain/ventral hippocampus (bregma −3.08 mm; Paxinos & Franklin 2012). Binding levels are represented using a pseudocolour interpretation of black and white film images in fmol/mg tissue equivalent. Representative autoradiograms for non‐specific binding (NSB) are shown (far right column). (b) Quantitative AMPA receptor binding. Levels of [^3^H]‐AMPA binding (fmol/mg tissue equivalent) are shown for prelimbic cortex (PrL), infralimbic cortex (IL), CA1 and CA2 regions of the ventral hippocampus combined (vCA1 + CA2), ventral hippocampus CA3 (vCA3), dorsal hippocampus CA1, CA2 and CA3 separately (dCA1, dCA2, dCA3), for each of the treatment groups (saline + saline control, saline + morphine‐primed reinstatement, MLA + saline control, MLA + morphine‐primed reinstatement). Data are presented as mean ± S.E.M. (*n* = 4–6). *Significantly different from saline control and from MLA‐pretreated, morphine‐primed condition, *P* < 0.05 (two‐way ANOVA)

Representative autoradiographs of [^3^H]‐AMPA binding are shown in Figure 3a. Labelling was particularly high in cortical areas and hippocampus, but very low in thalamic areas, as previously reported (Monaghan, Yao, & Cotman 1984). Quantitation of specific [^3^H]‐AMPA binding (Fig. 3b; Table S2) revealed a significant increase in [^3^H]AMPA binding density after morphine reinstatement in the CA1/CA2 subregions of the ventral hippocampus: (17.1 ± 3.3%; *P* < 0.05; *n* = 4–6/treatment group). There was no significant difference in vCA3 or any other region analysed.

Pretreatment with MLA before morphine‐primed reinstatement resulted in significantly lower binding in the CA1/CA2 subregion of the ventral hippocampus compared with values from mice that received saline before the morphine priming dose (Treatment × Behaviour interaction *F*(_1,15_) = 5.4, *P* < 0.05; Fig. 3b; Table S2). MLA pretreatment of saline controls did not affect [^3^H]‐AMPA binding, and there were no significant differences between treatment groups in any other region examined.

### Effect of intracerebral administration of methyllycaconitine on reinstatement of morphine‐conditioned place preference in rats

To evaluate the central locus of action of MLA in attenuating reinstatement of morphine‐CPP, local administration of MLA was undertaken. Because this approach is more appropriate for use in rats, it was necessary to first replicate the inhibition by systemic MLA of morphine‐primed reinstatement of CPP in this species. Male Wistar rats underwent conditioning (morphine 5 mg/kg s.c), extinction and then received either saline or a priming dose of morphine (2.5 mg/kg s.c) immediately before the reinstatement trial. MLA (4 mg/kg s.c) or saline was administered 20 minutes prior to the morphine‐priming dose. Drug doses were based on previous experience (Cordery *et al*. 2014) and literature precedent (Markou & Paterson 2001; Tzschentke 2007; Aguilar *et al*. 2009; Liu 2014).

**Figure 4:**
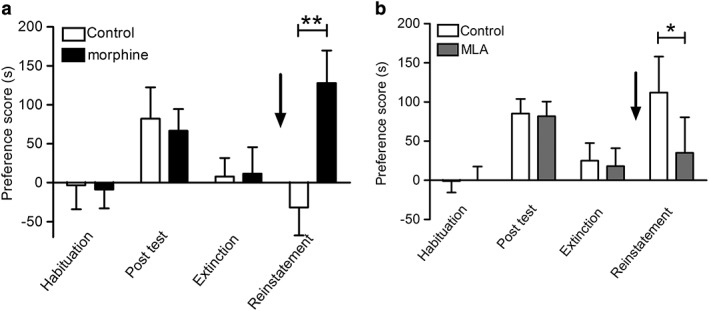
Morphine‐CPP in Wistar rats and effect of MLA on reinstatement. (a) Rats were divided into two groups, both groups underwent acquisition and extinction of morphine‐CPP (5 mg/kg morphine) as described win the Methods. Following extinction, one group received a priming dose of morphine (2.5 mg/kg) while the other group received saline (arrow). They were tested for reinstatement of morphine‐CPP. Data are presented as mean ± S.E.M. preference scores (*n* = 26). Morphine‐primed group was significantly different from saline controls, **P < 0.01 (one‐way ANOVA and Benjamini–Hochberg test for multiple comparisons). (b) Rats were divided into two groups, both groups underwent acquisition and extinction of morphine‐CPP as aforementioned. Following extinction, both groups received a priming dose of morphine (2.5 mg/kg), but one group received MLA (4 mg/kg) prior to the priming dose while the other group received saline (arrow). They were tested for reinstatement of morphine‐CPP. Data are presented as mean ± S.E.M. preference scores (*n* = 26). Saline‐pretreated morphine‐primed group was significantly different from morphine‐primed group that received MLA, *P < 0.05 (one‐way ANOVA and Benjamini–Hochberg test for multiple comparisons)

There was a significant acquisition of morphine‐CPP that was extinguished and reinstated in response to morphine (*P =* 0.007) but not saline (*P* = 0.34; Fig. 4a). Systemic MLA significantly inhibited the reinstatement of morphine‐primed CPP (Fig. 4b). There was no significant effect of treatment (*F*(_1,49_) = 0.70, *P =* 0.408) but a significant effect of test (F(_4,176_) = 3.96, *P =* 0.004, *n* = 26/treatment group). *Post hoc* pairwise comparisons revealed that only animals pretreated with saline prior to the morphine priming dose significantly reinstated (saline preference score at extinction: 25 ± 23 seconds versus 112 ± 46 seconds at reinstatement (*P =* 0.007); MLA preference score at extinction: 18 ± 23 seconds versus 35 ± 46 seconds at reinstatement, *P* = 0.667, *n* = 26/treatment group). The time spent in the drug paired side was significantly different between the two treatments (*P* = 0.024).

**Figure 5:**
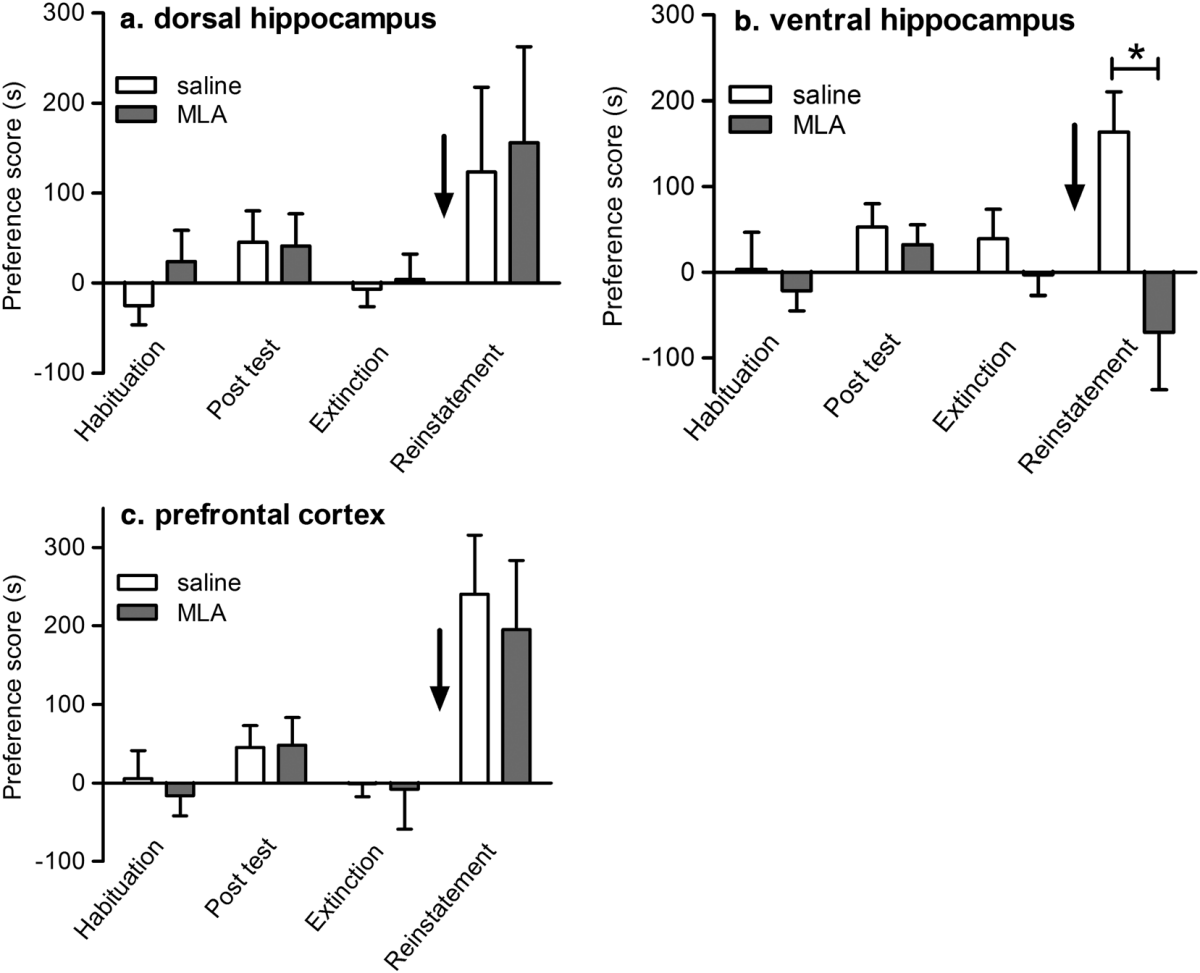
Effect of intracerebral administration of MLA on reinstatement of morphine‐CPP in rats. Rats acquired morphine‐CPP that was extinguished prior to the implantation of bilateral intracerebral cannulae in the dorsal hippocampus (a), ventral hippocampus (b) or prefrontal cortex (c). Animals then received either saline or MLA (6.75 μg/hemisphere) prior to a priming dose of morphine (2.5 mg/kg) (arrows) and testing for reinstatement of morphine‐CPP. Data are presented as mean ± S.E.M. preference scores (*n* = 8). In ventral hippocampus, saline pretreated morphine‐primed group was significantly different from morphine‐primed group that received MLA, *P < 0.05 (one‐way ANOVA and Benjamini–Hochberg test for multiple comparisons)

To test the effect of local delivery of MLA on morphine‐primed reinstatement of CPP, rats underwent acquisition and extinction of morphine‐CPP. Bilateral cannulae were then implanted in the dorsal hippocampus, ventral hippocampus, or mPFC. One week post surgery, animals received a bilateral infusion of saline or MLA (6.75 μg/hemisphere) 15 minutes prior to a morphine priming dose (2.5 mg/kg s.c) and free access to the CPP compartments to determine any preference. This dose of MLA, delivered intracerebrally, has been reported to be effective in other behavioural models (Addy, Nakijama, & Levin 2003; Nott & Levin 2006). Prior to surgery, there was a significant effect of morphine‐CPP (*F*(_2,94_) = 3.38, *P =* 0.0381) but no significant effect of treatment group (*F*(_1,47_) = 0.03, *P =* 0.8657, *n* = 25/treatment group), indicating acquisition and extinction of morphine‐CPP with no difference between the groups that would subsequently receive local MLA or saline infusions (Fig. 5). Animals that received saline intracerebrally prior to the priming dose of morphine showed robust reinstatement of morphine‐CPP (Fig. 5). Reinstatement was not affected by prior infusion of MLA into the dorsal hippocampus (*P =* 0.82, *n* = 8, Fig. 5a) or mPFC (*P =* 0.70, *n* = 8, Fig. 5c). However, MLA infused into the ventral hippocampus abolished morphine‐primed reinstatement of CPP (*P =* 0.012, *n* = 8, saline preference score at reinstatement: 164 ± 47 seconds versus MLA preference score at reinstatement: −70 ± 67 seconds; Fig. 5b). Thus, MLA delivered into the ventral hippocampus, but not the dorsal hippocampus or mPFC, completely blocks morphine‐primed reinstatement of morphine‐CPP.

## Discussion

This study corroborates and extends a previous report (Feng *et al*. 2011) that blockade of α7 nAChRs attenuates reinstatement of morphine‐primed CPP in mice. Here, we have reproduced this finding in a different strain of mouse (C57BL/6, compared with BalbC used by Feng *et al*. (2011)) and extended it to Wistar rats. Further, we have shown that α7 nAChR blockade by systemic delivery of MLA is specific in only inhibiting the reinstatement phase of the CPP paradigm, and that this action is reproduced by MLA delivered into the ventral hippocampus (but not the dorsal hippocampus or mPFC). This locus of action of MLA accords with increases in AMPA receptor binding in CA1/CA2 regions of the ventral hippocampus in response to morphine‐primed reinstatement that are also attenuated by systemic MLA.

CPP provides a model of Pavlovian associative learning, in which the unconditional stimulus (drug) is paired with a conditional stimulus (environmental context) (Bardo & Bevins 2000; Tzschentke 2007; Aguilar *et al*. 2009). The ability of rodents to rapidly learn to positively associate a distinctive chamber of the CPP apparatus with the unconditioned stimulus, measured as the drug‐free preference score, reflects the incentive salience of the drug. This associative learning has face validity for human drug addiction in which strong associations are formed with the context of drug taking (Napier, Herrold, & de Wit 2013). Other aspects of drug dependence, maintenance, reconsolidation and extinction, are also modelled in the CPP paradigm. The endurance of the associative learning beyond extinction is demonstrated by the ready reinstatement of CPP in response to a trigger, in this case, a priming drug dose (Aguilar *et al*. 2009). Reinstatement provides a useful model relevant to relapse in abstinent addicts (Napier *et al*. 2013). Relapse is a major social problem and interventions that target this aspect of drug dependency would be advantageous.

The selective effect of MLA on morphine‐primed reinstatement is robust, as demonstrated in both mice and rats and via two modes of delivery (systemic and intracerebral). The data suggest that α7 nAChRs contribute specifically to this phase of the addictive drug cycle. Interestingly, a selective effect for α7 nAChRs in cue‐induced reinstatement of nicotine self‐administration has also been reported (Liu 2014). More recently, increasing kynurenic acid (a negative modulator of α7 nAChRs) was found to attenuate both drug‐induced and cue‐induced reinstatement of nicotine and cocaine self‐administration in squirrel monkeys (Secci *et al*. 2017). Evidence that this effect was mediated by α7 nAChRs was provided by the opposing action of a positive allosteric modulator. Together, these studies support a role for α7 nAChRs more generally in circuitry responsible for the retrieval of drug and context associated memories. Basal forebrain cholinergic activity appears necessary for cue‐induced relapse (Pitchers *et al*. 2017), and α7 nAChRs may mediate, at least in part, this cholinergic signalling.

The hippocampus is a major target of cholinergic projection neurons from the basal forebrain. α7 nAChRs are highly expressed in the hippocampus where they can modulate glutamate‐mediated synaptic plasticity (Yakel 2014). Synaptic strengthening is correlated with an increase in the number of synaptic AMPA (but not N‐methyl‐d‐aspartate) receptors (Kessels & Malinow 2009), and this may be reflected in the significant increase in [^3^H]‐AMPA binding localized to the CA1/CA2 area of the ventral hippocampus after morphine‐primed reinstatement. Inhibition of the increased [^3^H]‐AMPA binding by prior administration of MLA (Fig. 3) suggests that α7 nAChRs play a critical role in facilitating such synaptic changes. The ability of MLA delivered into the ventral hippocampus (but not other brain areas examined) to abolish morphine‐primed reinstatement links the behavioural response to α7 nAChR‐mediated mechanisms in this brain region. The ventral hippocampus is connected to circuitry related to emotion, stress and affect; connections to the nucleus accumbens shell, prefrontal cortex and amygdala give it a central role in motivational learning (Fanselow & Dong 2010).

With respect to nAChRs in addiction, most attention has focussed on the roles of nAChR subtypes in nicotine dependence. α4β2 nAChRs are critical in mediating the rewarding properties of nicotine (Picciotto *et al*. 1998; Pons *et al*. 2008) and the α4β2 nAChR‐selective antagonist dihydroβerythroidine reduces self‐administration (Watkins *et al*. 1999; Grottick *et al*. 2000), with a locus of action in the VTA (Corrigall *et al*. 1994). In contrast, pharmacological antagonism or genetic deletion of α7 nAChRs has been reported to have no effect on nicotine self‐administration (Grottick *et al*. 2000; Pons *et al*. 2008; Liu 2014) or acquisition of nicotine‐CPP (Walters *et al*. 2006), although other studies have shown an attenuation of intravenous (Markou & Paterson 2001) or intracranial nicotine self‐administration (Besson *et al*. 2012), without affecting the somatic signs of nicotine withdrawal (Markou & Paterson 2001). However, Liu (2014) found that systemic MLA (but not dihydroβerythroidine) selectively attenuated cue‐induced reinstatement of nicotine seeking in rats, without affecting the development of nicotine self‐administration. These findings suggest distinct roles for α4β2 and α7 nAChRs in nicotine reward and relapse, respectively, and resonate with the selective role for α7 nAChRs in the current study, in which acquisition, maintenance and reconsolidation of morphine‐CPP were unaffected by MLA.

In the present work, systemically administered MLA substantially attenuated morphine‐primed reinstatement of CPP whereas delivery of MLA into the ventral hippocampus completely abolished the response. This could reflect a bigger local dose effect intracerebrally that fully antagonized α7 nAChRs, whereas the systemic dose of 4 mg/kg might be suboptimal. Indeed Liu (2014) found a significant dose effect of MLA at 2.5 and 10 mg/kg for inhibition of reinstatement of cue‐induced nicotine self‐administration in rats. However, high doses of MLA run the risk of also antagonizing other nAChR subtypes (notably α6β2 nAChRs) because this antagonist is selective rather than specific for α7 nAChRs (Mogg *et al*. 2002). The negligible level of α6‐containing nAChRs in the hippocampus supports a specific action of MLA at α7 nAChRs in the ventral hippocampus. An alternative explanation of the greater efficacy of locally administered MLA is that α7 nAChRs may exert opposing influences in other brain regions such as the PFC (Bloem, Poorthuis, & Mansvelder 2014; Udakis *et al*. 2016) that temper the profound antagonism mediated by the ventral hippocampus.

This study provides evidence for a selective role for α7 nAChRs in the ventral hippocampus in the reinstatement of morphine‐primed CPP, which encourages further investigation of α7 nAChRs as potential targets for preventing opiate relapse. α7 nAChRs appear to play a similar role in nicotine reinstatement (Liu 2014) and have been implicated in cocaine reinstatement (Secci *et al*. 2017). Therefore, an α7 nAChR‐mediated mechanism may contribute to the reinstatement of drug seeking more generally in abstinent addicts.

## Supporting information


**Table S1.** Specific [^3^H]‐(+)‐MK801 binding to sections of mouse brain after reinstatement of morphine‐CPP, or saline–primed controls, with or without pre‐treatment with MLA (4 mg/kg s.c.). Sections were processed and analysed as described in the Methods (Kitchen *et al*. 1997). Data are the mean ± S.E.M. from 5‐6 animals. Cortical regions: Prelimbic (PrL), Infralimbic (IL), Motor (M1‐2), Cingulate (CgCx), Auditory (AuCx) and visual (ViCx); Caudate putamen (CPu), Accumbens shell (Acbs) and core (Acbc), Dorsal Hippocampus CA1‐3 (dCA1, dCA2, dCA3), Central Amygdala (CeA), Basolateral Amygdala (BLA), and Basomedial (BMA) nuclei of the Amygdala, Ventral Hippocampus CA1‐3 (vCA1, vCA2, vCA3), Ventral Tegmental Area (VTA).

**Table S2.** Specific [^3^H]‐AMPA binding to sections of mouse brain after reinstatement of morphine‐CPP, or saline–primed controls, with or without pre‐treatment with MLA (4 mg/kg s.c.). Sections were processed and analysed as described in the Methods (Kitchen *et al*. 1997). Data are the mean ± S.E.M. from 5‐6 animals. Cortical regions: Prelimbic (PrL), Infralimbic (IL), Motor (M1‐2), Cingulate (CgCx), Auditory (AuCx) and visual (ViCx); Caudate putamen (CPu), Accumbens core (Acbc) and shell (Acbs), Basomedial nuclei of the Amygdala (BMA), Central Amygdala (CeA), Basolateral Amygdala (BLA); Dorsal Hippocampus CA1‐3 (dCA1, dCA2, dCA3), and Ventral Hippocampus CA1‐3 (vCA1 + CA2, vCA3), Ventral Tegmental Area (VTA).

**Figure S1.** Morphine‐CPP in mice. The ability of morphine to induce CPP was compared with saline‐treated mice, in protocols analogous to the schematics in Figure 1. *A. Acquisition of morphine‐CPP*. Animals were habituated to the CPP apparatus on 2 consecutive days and pseudo‐randomly assigned to two groups with similar mean preference scores. Mice given morphine (10 mg/kg) or saline on 4 consecutive days, in alternate compartments, displayed a robust preference for the morphine‐paired compartment (***p<0.001, *n*=12/treatment group). Mice that received saline in both compartments displayed no preference. *B. Extinction and reinstatement of morphine‐CPP*. Mice were pseudo‐randomly assigned to two groups and both groups acquired morphine‐CPP, which was then extinguished following 4 days of pairing saline injections with the previously morphine‐paired compartment (see Figure 1C). One group (black bars) then received a priming dose of morphine (5 mg/kg) and displayed a robust reinstatement of preference for the morphine‐paired compartment. The control group that received saline at this stage did not reinstate (morphine‐primed preference score: 150.0±29.1 seconds, saline‐primed preference score: ‐13.7±56.6 seconds in previously morphine‐paired compartment, ***p<0.001, *n*=10‐12/treatment group).

**Figure S2.** A schematic diagram illustrating the distribution of bilateral injection cannula placements. To verify cannula placements, at the end of each experiment rats were killed by rising CO2 asphyxiation and 0.5 μl brilliant blue dye was infused via each cannula. Coronal sections were taken from rats that had previously been given saline prior to morphine‐primed CPP reinstatement (A) and rats given MLA prior to morphine‐primed CPP reinstatement (B). Regions of dye infusion were compared with a brain atlas (Paxinos & Watson 2007). Black dots indicate cannulae placements in individual animals. Smaller red dots indicate target coordinates.
