# Supplementary material for: Inhibition of alpha7 nicotinic receptors in the ventral hippocampus selectively attenuates reinstatement of morphine‐conditioned place preference and associated changes in AMPA receptor binding
Source: Addict Biol. 2018 Apr 17;24(4):590–603. doi: 10.1111/adb.12624 (PMC6563460; doi:10.1111/adb.12624)
Supplement: Supplementary file 1 — Table S1. Specific [3H]‐(+)‐MK801 binding to sections of mouse brain after reinstatement of morphine‐CPP, or saline–primed controls, with or without pre‐treatment with MLA (4 mg/kg s.c.). Sections were processed and analysed as described in the Methods (Kitchen et al. 1997). Data are the mean ± S.E.M. from 5‐6 animals. Cortical regions: Prelimbic (PrL), Infralimbic (IL), Motor (M1‐2), Cingulate (CgCx), Auditory (AuCx) and visual (ViCx); Caudate putamen (CPu), Accumbens shell (Acbs) and core (Acbc), Dorsal Hippocampus CA1‐3 (dCA1, dCA2, dCA3), Central Amygdala (CeA), Basolateral Amygdala (BLA), and Basomedial (BMA) nuclei of the Amygdala, Ventral Hippocampus CA1‐3 (vCA1, vCA2, vCA3), Ventral Tegmental Area (VTA). [file ADB-24-590-s003.pdf]

Distance from Bregma	Brain Region	Specific [^{3}H]-(+)-MK801 binding (fmol / mg tissue equivalent) — Pre-treatment and priming treatment at reinstatement stage				p value (i) sal/morphine vs sal/sal	p value (ii) MLA/morphine vs sal/morphine
		Saline, Saline	Saline, Morphine	MLA, Saline	MLA, Morphine		
1.94mm	PrL	407.2 ± 16.5	426.2 ± 35.4	395.3 ± 44.6	428.0 ± 27.4	ns	ns
	IL	387.3 ± 24.6	425.5 ± 38.2	396.5 ± 34.3	421.9 ± 33.5	ns	ns
	M1	350.0 ± 39.7	395.6 ± 40.0	307.3 ± 45.2	378.5 ± 23.5	ns	ns
	M2	359.0 ± 33.7	407.5 ± 35.0	323.6 ± 50.8	377.1 ± 22.2	ns	ns
	CgCx	388.8 ± 32.3	421.7 ± 31.8	370.4 ± 32.2	414.2 ± 17.1	ns	ns
1.42mm	CPu	232.3 ± 14.6	243.0 ± 7.3	246.9 ± 24.6	208.4 ± 21.0	ns	ns
	AcbC	267.3 ± 8.2	235.8 ± 15.8	256.3 ± 28.0	231.4 ± 22.6	ns	ns
	AcbS	249.2 ± 25.0	204.0 ± 24.4	254.8 ± 28.2	224.3 ± 32.7	ns	ns
-1.22mm	BMA	287.7 ± 23.3	314.2 ± 43.3	280.3 ± 24.1	312.4 ± 19.7	ns	ns
	CeA	270.3 ± 16.9	270.5 ± 31.8	219.8 ± 30.2	299.0 ± 11.0	ns	ns
	BLA	336.8 ± 11.2	331.1 ± 29.9	308.6 ± 29.5	343.5 ± 26.2	ns	ns
	dCA1	662.3 ± 35.6	701.4 ± 53.0	549.0 ± 67.9	677.4 ± 59.3	ns	ns
	dCA2	661.3 ± 36.5	692.5 ± 51.5	546.0 ± 67.6	677.7 ± 54.7	ns	ns
	dCA3	383.2 ± 41.9	431.5 ± 56.1	323.6 ± 39.3	459.9 ± 57.0	ns	ns
	vCA1 + CA2	746.5 ± 34.0	805.7 ± 23.0	689.7 ± 53.6	699.0 ± 66.4	ns	ns
	vCA3	450.0 ± 58.9	460.3 ± 40.8	393.8 ± 43.0	465.3 ± 27.1	ns	ns
	VTA	67.3 ± 23.6	49.8 ± 10.8	58.7 ± 18.9	68.1 ± 20.9	ns	ns
	AuCx	371.1 ± 14.7	392.3 ± 17.5	343.3 ± 32.9	362.9 ± 33.9	ns	ns
	ViCx	322.7 ± 14.1	366.7 ± 17.7	287.1 ± 40.6	354.3 ± 32.0	ns	ns

Table S1 Specific [^{3}H]-(+)-MK801 binding to sections of mouse brain after reinstatement of morphine CPP, or saline – primed controls, with or without pre-treatment with MLA (4 mg/kg s.c.). Sections were processed and analysed as described in the Methods (Kitchen et al., 1997). Data are the mean ± S.E.M. from 5-6 animals. Cortical regions: Prelimbic (PrL), Infralimbic (IL), Motor (M1-2), Cingulate (CgCx), Auditory (AuCx) and visual (ViCx); Caudate putamen (CPu), Accumbens shell (Acbs) and core (Acbc), Dorsal Hippocampus CA1-3 (dCA1, dCA2, dCA3), Central Amygdala (CeA), Basolateral Amygdala (BLA), and Basomedial (BMA) nuclei of the Amygdala, Ventral Hippocampus CA1-3 (vCA1, vCA2, vCA3), Ventral Tegmental Area (VTA).
