# Supplementary material for: Inhibition of alpha7 nicotinic receptors in the ventral hippocampus selectively attenuates reinstatement of morphine‐conditioned place preference and associated changes in AMPA receptor binding
Source: Addict Biol. 2018 Apr 17;24(4):590–603. doi: 10.1111/adb.12624 (PMC6563460; doi:10.1111/adb.12624)
Supplement: Supplementary file 2 — Table S2. Specific [3H]‐AMPA binding to sections of mouse brain after reinstatement of morphine‐CPP, or saline–primed controls, with or without pre‐treatment with MLA (4 mg/kg s.c.). Sections were processed and analysed as described in the Methods (Kitchen et al. 1997). Data are the mean ± S.E.M. from 5‐6 animals. Cortical regions: Prelimbic (PrL), Infralimbic (IL), Motor (M1‐2), Cingulate (CgCx), Auditory (AuCx) and visual (ViCx); Caudate putamen (CPu), Accumbens core (Acbc) and shell (Acbs), Basomedial nuclei of the Amygdala (BMA), Central Amygdala (CeA), Basolateral Amygdala (BLA); Dorsal Hippocampus CA1‐3 (dCA1, dCA2, dCA3), and Ventral Hippocampus CA1‐3 (vCA1 + CA2, vCA3), Ventral Tegmental Area (VTA). [file ADB-24-590-s004.pdf]

Distance from Bregma	Brain Region	Specific [3H]-AMPA binding fmol/mg tissue equivalent)				p value (i)	p value (ii)
		Pre-treatment and priming treatment at reinstatement stage				sal/morphine vs sal/sal	MLA/morphine vs sal/morphine
		Saline, Saline	Saline, Morphine	MLA, Saline	MLA, Morphine		
1.94mm	PrL	216.9 ± 08.3	226.7 ± 23.3	216.3 ±9.9	177.9 ± 11.0	ns	ns
	IL	208.9 ± 17.5	199.3 ± 24.1	220.6 ± 8.3	184.1 ± 18.6	ns	ns
	M1	184.8 ± 07.0	204.3 ± 10.3	187.5 ± 13.1	179.5 ± 19.7	ns	ns
	M2	202.0 ± 04.8	221.2 ± 12.1	205.0 ± 7.5	194.3 ± 17.5	ns	ns
	CgCx	211.6 ± 03.6	228.5 ± 21.7	215.9 ± 13.3	189.3 ± 14.3	ns	ns
1.42mm	CPu	131.1 ± 01.3	122.2 ± 7.00	134.9 ± 4.0	140.3 ± 11.1	ns	ns
	AcbC	188.2 ± 07.2	190.5 ± 11.6	163.3 ± 18.7	188.7 ± 18.8	ns	ns
	AcbS	168.1 ± 17.4	148.4 ± 16.5	149.9 ± 20.2	171.8 ± 18.5	ns	ns
-1.22mm	BMA	195.1 ± 11.5	185.3 ± 5.00	150.8 ± 7.7	189.6 ± 21.3	ns	ns
	CeA	180.7 ± 17.2	187.6 ± 9.90	154.3 ± 5.4	181.6 ± 19.9	ns	ns
	BLA	203.3 ± 15.1	200.5 ± 8.60	192.5 ± 9.8	193.8 ± 17.7	ns	ns
	dCA1	363.6 ± 20.5	352.3 ± 54.0	347.3 ± 24.8	360.2 ± 40.0	ns	ns
	dCA2	351.8 ± 21.0	314.1 ± 49.1	361.4 ± 19.2	328.9 ± 29.5	ns	ns
	dCA3	202.6 ± 20.4	160.2 ± 27.2	169.9 ± 15.9	178.0 ± 24.3	ns	ns
	vCA1+CA2	387.9 ± 18.7	454.1 ± 13.0	369.3 ± 24.9	350.0 ± 39.9	0.03	0.02
	vCA3	255.8 ± 23.8	304.1 ± 20.8	254.4 ± 17.8	275.6 ± 16.6	ns	ns
	VTA	023.2 ± 03.2	50.00 ± 11.3	41.7 ± 6.9	38.1 ± 4.8	ns	ns
	AuCx	178.8 ± 12.5	204.8 ± 11.9	170.0 ± 10.9	157.7 ± 11.0	ns	ns
	ViCx	162.5 ± 16.7	203.7 ± 15.3	146.8 ± 7.9	165.8 ± 12.3	ns	ns

Table S2 Specific [^{3}H]-AMPA binding to sections of mouse brain after reinstatement of morphine-CPP, or saline–primed controls, with or without pre-treatment with MLA (4 mg/kg s.c.). Sections were processed and analysed as described in the Methods (Kitchen et al., 1997). Data are the mean ± S.E.M. from 5-6 animals. Cortical regions: Prelimbic (PrL), Infralimbic (IL), Motor (M1-2), Cingulate (CgCx), Auditory (AuCx) and visual (ViCx); Caudate putamen (CPu), Accumbens core (Acbc) and shell (Acbs), Basomedial nuclei of the Amygdala (BMA), Central Amygdala (CeA), Basolateral Amygdala (BLA); Dorsal Hippocampus CA1-3 (dCA1, dCA2, dCA3), and Ventral Hippocampus CA1-3 (vCA1 + CA2, vCA3), Ventral Tegmental Area (VTA).
