# Supplementary material for: Inhibition of alpha7 nicotinic receptors in the ventral hippocampus selectively attenuates reinstatement of morphine‐conditioned place preference and associated changes in AMPA receptor binding
Source: Addict Biol. 2018 Apr 17;24(4):590–603. doi: 10.1111/adb.12624 (PMC6563460; doi:10.1111/adb.12624)
Supplement: Supplementary file 3 — Figure S1. Morphine‐CPP in mice. The ability of morphine to induce CPP was compared with saline‐treated mice, in protocols analogous to the schematics in Figure 1. A. Acquisition of morphine‐CPP. Animals were habituated to the CPP apparatus on 2 consecutive days and pseudo‐randomly assigned to two groups with similar mean preference scores. Mice given morphine (10 mg/kg) or saline on 4 consecutive days, in alternate compartments, displayed a robust preference for the morphine‐paired compartment (***p<0.001, n=12/treatment group). Mice that received saline in both compartments displayed no preference. B. Extinction and reinstatement of morphine‐CPP. Mice were pseudo‐randomly assigned to two groups and both groups acquired morphine‐CPP, which was then extinguished following 4 days of pairing saline injections with the previously morphine‐paired compartment (see Figure 1C). One group (black bars) then received a priming dose of morphine (5 mg/kg) and displayed a robust reinstatement of preference for the morphine‐paired compartment. The control group that received saline at this stage did not reinstate (morphine‐primed preference score: 150.0±29.1 seconds, saline‐primed preference score: ‐13.7±56.6 seconds in previously morphine‐paired compartment, ***p<0.001, n=10‐12/treatment group). [file ADB-24-590-s001.pdf]

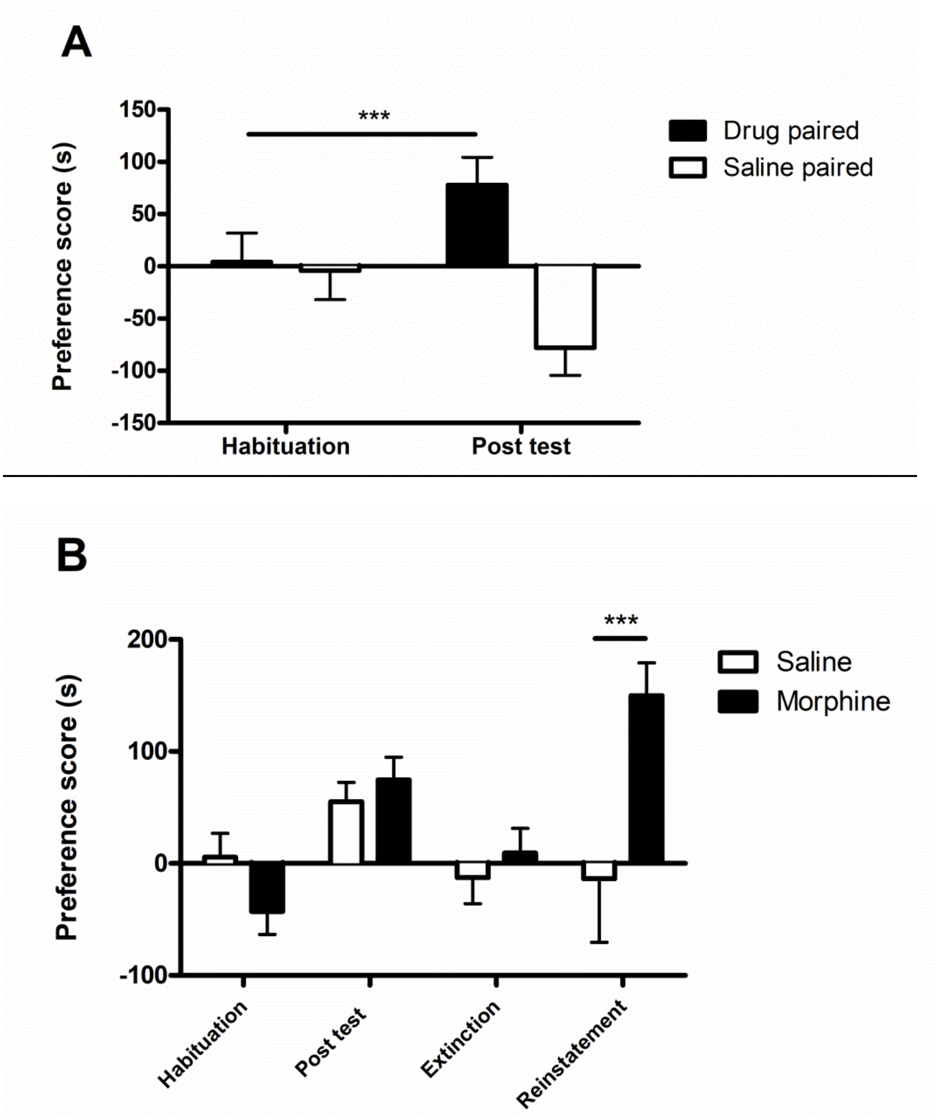

Figure S1 Morphine-CPP in mice.

The ability of morphine to induce CPP was compared with saline-treated mice, in protocols analogous to the schematics in Figure 1.

A. Acquisition of morphine-CPP. Animals were habituated to the CPP apparatus on 2 consecutive days and pseudo-randomly assigned to two groups with similar mean preference scores. Mice given morphine (10 mg/kg) or saline on 4 consecutive days, in alternate compartments, displayed a robust preference for the morphine-paired compartment (***p<0.001, n=12/treatment group). Mice that received saline in both compartments displayed no preference.

B. Extinction and reinstatement of morphine-CPP. Mice were pseudo-randomly assigned to two groups and both groups acquired morphine-CPP, which was then extinguished following 4 days of pairing saline injections with the previously morphine-paired compartment (see Figure 1C). One group (black bars) then received a priming dose of morphine (5 mg/kg) and displayed a robust reinstatement of preference for the morphine-paired compartment. The control group that received saline at this stage did not reinstate (morphine-primed preference score: 150.0±29.1 s, saline-primed preference score: -13.7±56.6 s in previously morphine-paired compartment, ***p<0.001, n=10-12/treatment group).
