# Supplementary material for: Inhibition of alpha7 nicotinic receptors in the ventral hippocampus selectively attenuates reinstatement of morphine‐conditioned place preference and associated changes in AMPA receptor binding
Source: Addict Biol. 2018 Apr 17;24(4):590–603. doi: 10.1111/adb.12624 (PMC6563460; doi:10.1111/adb.12624)
Supplement: Supplementary file 4 — Figure S2. A schematic diagram illustrating the distribution of bilateral injection cannula placements. To verify cannula placements, at the end of each experiment rats were killed by rising CO2 asphyxiation and 0.5 μl brilliant blue dye was infused via each cannula. Coronal sections were taken from rats that had previously been given saline prior to morphine‐primed CPP reinstatement (A) and rats given MLA prior to morphine‐primed CPP reinstatement (B). Regions of dye infusion were compared with a brain atlas (Paxinos & Watson 2007). Black dots indicate cannulae placements in individual animals. Smaller red dots indicate target coordinates. [file ADB-24-590-s002.pdf]

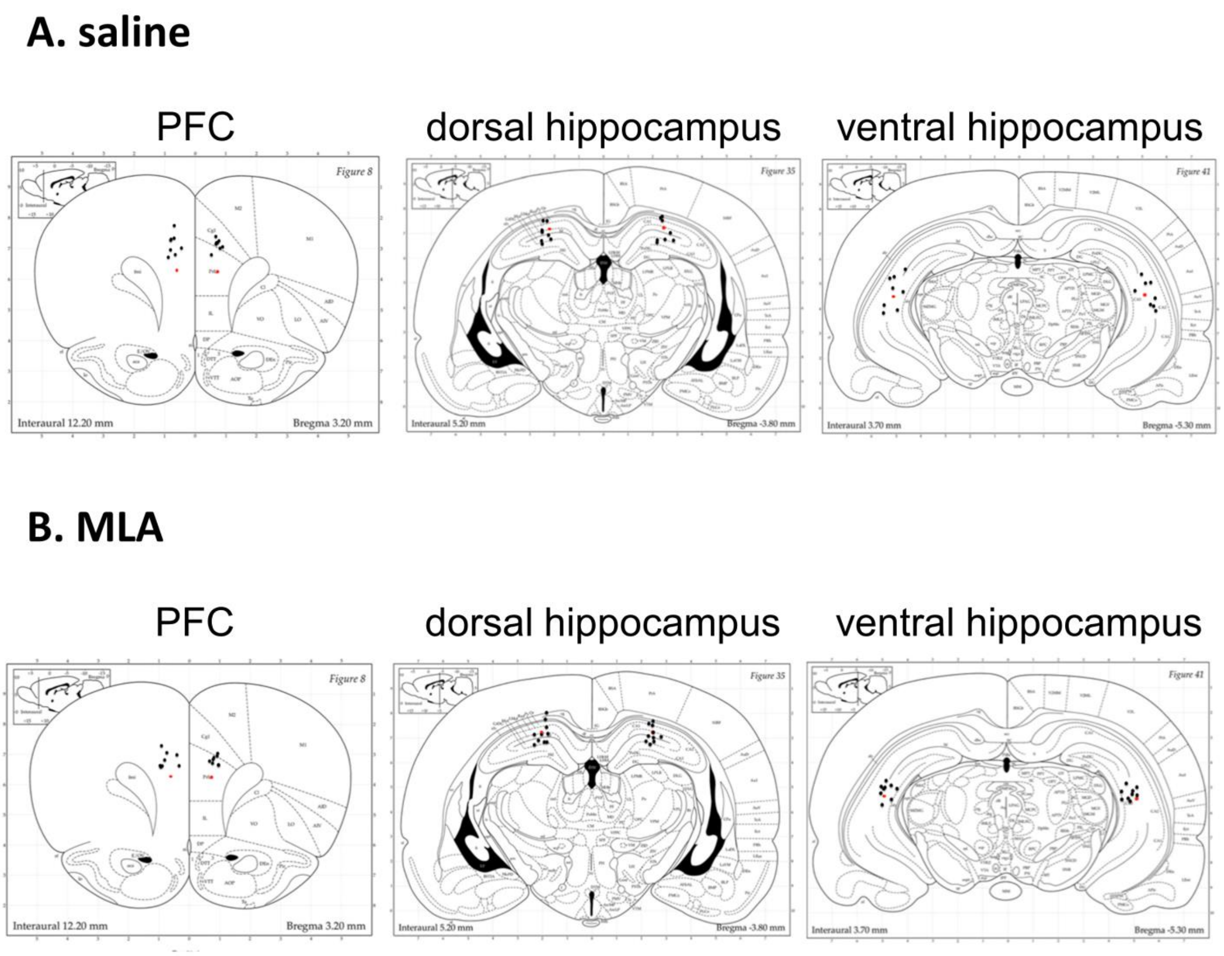

Figure S2 A schematic diagram illustrating the distribution of bilateral injection cannula placements

To verify cannula placements, at the end of each experiment rats were killed by rising CO_2 asphyxiation and 0.5 µl brilliant blue dye was infused via each cannula. Coronal sections were taken from rats that had previously been given saline prior to morphine-primed CPP reinstatement (A) and rats given MLA prior to morphine-primed reinstatement (B). Regions of dye infusion were compared with a brain atlas (Paxinos and Watson, 2007). Black dots indicate cannulae placements in individual animals. Smaller red dots indicate target coordinates.
